# Unaffected Memory and Inhibitory Functioning Several Weeks Postpartum in Women with Pregnancy Complicated by Preeclampsia

**DOI:** 10.3390/bs11040055

**Published:** 2021-04-18

**Authors:** Ilona Papousek, Elisabeth M. Weiss, Manfred G. Moertl, Karin Schmid-Zalaudek, Edina Krenn, Verena Lessiak, Helmut K. Lackner

**Affiliations:** 1 Biological Psychology Unit, Department of Psychology, University of Graz, 8010 Graz, Austria; ilona.papousek@uni-graz.at (I.P.); edina.krenn@gmail.com (E.K.); 2 Clinical Psychology Unit, Department of Psychology, University of Innsbruck, 6020 Innsbruck, Austria; Elisabeth.Weiss@uibk.ac.at; 3 Clinical Center, Department of Obstetrics and Gynecology, 9020 Klagenfurt, Austria; manfredmoertl@gmx.at (M.G.M.); v.lessiak@gmail.com (V.L.); 4 Otto Loewi Research Center, Division of Physiology, Medical University of Graz, 8010 Graz, Austria; karin.schmid@medunigraz.at

**Keywords:** preeclampsia, pregnancy, memory, inhibitory control, stress

## Abstract

Several studies reported impaired cognitive functioning after pregnancy complicated by preeclampsia. The present study examined cognitive and executive functioning in women with preeclampsia at a time at which immediate effects of gestation have resolved, brain damage due to other risk factors have not yet manifested, and impairments may thus primarily occur as a result of the huge stress induced by the potentially life threatening condition. Verbal learning/memory (California Verbal Learning Test) and inhibitory functioning (Mittenecker Pointing Test) of 35 women with preeclampsia and 38 women with uncomplicated pregnancy were followed over five measurement time points during the period from 16 to 48 weeks postpartum. A further control group comprised 40 women with no history of recent pregnancy. The groups did not differ in their verbal learning/memory performance. Higher levels of currently experienced everyday-life stress were associated with poorer inhibitory control/greater stereotypy in responding, but this effect was not directly connected with pregnancy complications. Taken together, the findings do not indicate rapid-onset cognitive impairment after preeclampsia, brought about by its extremely stressful nature or other factors that take effect during gestation. Deficits observed in later life may develop on a long-term basis through late-diagnosed hypertension and unfavorable lifestyle factors. The large time window in which exaggerated cognitive decline can be prevented or mitigated should be utilized for the control of risk factors and interventions to improve lifestyle where appropriate.

## 1. Introduction

Pregnancy and childbirth can be a source of stress for many women. Up to 20–48% of women describe their childbirth as a “traumatic” experience [1]. This applies all the more to pregnancies with complications that imply a threat to the woman’s health, her baby, or both [2,3]. One of these is preeclampsia. Preeclampsia is a hypertensive disorder of pregnancy characterized by the sudden onset of hypertension with either proteinuria, end-organ dysfunction, or both, after the 20th week of gestation in a previously normotensive woman [4]. It is potentially life threatening for the mother and fetus, and is typically associated with preterm birth.

The exposure to a highly stressful event may impact cognitive and executive functioning, with verbal memory and inhibitory control among the most affected functions [5,6,7]. In the case of a continuing stress experience, impairments are supposed to be mediated by functional changes such as decreased hippocampal excitability and altered sensitivity of prefrontal regions via the action of corticosteroids and catecholamines [8,9]. High levels of chronic stress are associated with impaired inhibitory functioning also in the absence of trauma and affective psychopathology [10,11]. Exposure to severe stress for very long periods of time have been linked to neurotoxic effects that may even result in structural changes such as hippocampal and prefrontal cortical neuronal loss and dendritic atrophy [8,12].

Pregnancy as such may affect memory functions, but impairments are subtle and seem to turn up in tests that place relatively high demands on effortful processing only, such as free recall [13]. Subtle deficits present during pregnancy may extend into the early postpartum period [13,14]. While some changes in the brain structure during pregnancy have been reported [15], there is limited evidence on their primary causes and the roles they may play in cognitive impairments during pregnancy [16]. In addition to fluctuating hormone levels, an important factor may be the manifold strains during gestation and childbirth, which affect the brain on their own through the effects of chronic stress [12,17]. Severe pregnancy complications such as preeclampsia may then potentiate these effects. Previous studies reported that declines in memory functions primarily occurred with high levels of chronic stress, manifested, for instance, in symptoms of depression or anxiety [17,18].

In women with a history of preeclampsia, several studies have observed impaired memory and executive functioning many years after gestation, that is, when the huge stress caused by the complicated pregnancy is long gone [19,20]. However, a large, well-controlled prospective study came to the conclusion that preeclampsia does not seem to be independently associated with cognitive impairment in later life. Rather, deficits in middle and older age seem to be attributed to factors that affect the brain on a long-term basis and are more prevalent in women with former preeclampsia, such as hypertension, poor eating habits, and low education [21]. In even older women, an epidemiological study found a history of preeclampsia to be associated with a greater risk of vascular dementia over and above those general risk factors [22], while other studies did not share this conclusion [23,24].

The present study seeks to add another vital component to this evidence of possible changes in cognitive functioning after preeclampsia. Its basic idea is that if memory and inhibitory functioning in women with preeclampsia are affected due to the highly stressful nature of the pregnancy complication, impairments should already show in the weeks and months following childbirth, that is, already at a time when serious brain damage brought about by classic risk factors or sustained neurovascular dysfunction has not yet manifested. To date, only a few studies tested relevant cognitive or executive functions in that time period, and so far did not provide a clear picture. Two days postpartum, Rana et al. [25] found no differences in verbal learning and memory between 15 women with preeclampsia and 15 women with uncomplicated pregnancies. Both groups seemed impaired compared to normative standards of nonpregnant women at the same age. In a study of Brussé et al. [26] three to seven months postpartum (median at three months), women with former preeclampsia (*n* = 10) showed poorer verbal learning and memory scores compared to women with uncomplicated pregnancies. No differences were observed in tests tapping executive functioning. Baecke et al. [27] tested 27 women with preeclampsia and 19 women with uncomplicated pregnancy 16 to 18 months postpartum and did not find differences in verbal learning and memory.

Thus, in the present study, we systematically followed the verbal learning and memory and inhibitory functioning of women with preeclampsia and women with uncomplicated pregnancies over a period from 16 to 48 weeks postpartum. Five measurement time points allowed us to observe the temporal progress of functioning in this period. Unlike from most other studies in the field, the effects of comorbidities were ruled out by testing only women who did not have eclampsia or other apparent neurological symptoms during gestation or a history of a psychiatric disorder at study entry. To be able to better classify their functioning, the performance of women with former preeclampsia was also compared to that of a control group of women with no recent pregnancy. Objective and particularly suitable tests were used, which are also sensitive in the non-pathological range and can also capture subtle changes in the functions of interest. To demarcate the potential effects, the current levels of experienced everyday-life stress at the time of testing and distress in terms of depressive symptoms were quantitatively assessed. Due to the sparse and heterogeneous evidence from relevant previous studies and the diversity of methods employed in those studies, the study was primarily explorative in nature rather than based on a specific a-priori hypothesis.

## 2. Materials and Methods

### 2.1. Study Sample

The study sample comprised 35 women with preeclampsia, 38 women with uncomplicated pregnancies, and a control group of 40 women who were non-pregnant during the last three years. Eligible mothers were preselected on the basis of the following criteria taken from their medical records, and were invited to participate in the study 13–15 weeks after childbirth.

Preeclampsia was confirmed using the recommendations of the American College of Obstetricians and Gynecologists’ Task Force on Hypertension in Pregnancy [28]. The inclusion criteria were: Systolic blood pressure ≥ 140 mmHg and/or diastolic blood pressure ≥ 90 mmHg, presenting at ≥20 weeks gestation and returning to normotensive values within 12 weeks postpartum, blood pressure measured twice and at least 4 h apart. Proteinuria: either protein ≥ 300 mg per 24 h urine collection or protein/creatinine ratio ≥ 0.3, or protein ≥ 30 mg/dL, or 1+ on urine dipstick.

Severe preeclampsia was defined as above, but one of the following had to be present and proteinuria was not required: systolic blood pressure ≥160 mmHg, measured twice at least 15 min apart; diastolic blood pressure ≥ 110 mmHg, measured twice at least 15 min apart; thrombocytopenia: platelet count < 100,000/µL; impaired liver function: AST or ALT ≥ 70 units/L or twice the normal concentration; renal insufficiency: serum creatinine ≥ 1.1 mg/dL or doubled from baseline values; or pulmonary edema. None of the pregnancies was complicated by eclampsia. No women had experienced perinatal death. Further exclusion criteria were: multiple gestation, diabetes mellitus, renal disease, chronic hypertension, antiphospholipid antibody syndrome, kidney transplant, hypothyroidism, thyroid antibodies, preexisting cardiovascular problems, seizures, history of psychiatric disorders. Appropriate education (minimum school-leaving qualification, nine educational years) and language competence (native German or German B2) were required for inclusion. Participants with uncomplicated pregnancies had singleton pregnancies with term delivery. Data in the two pregnancy groups were collected at weeks 16, 24, 32, 40, and 48 postpartum (±1 w).

In the no recent-pregnancy control group, data were obtained at one measurement time point only.

The study was performed in accordance with the Declaration of Helsinki and was approved by the ethics committee of the Medical University Graz, Austria (No. 27-515 ex 14/15) and the ethics committee Carinthia, Austria (No. A16/15). Written informed consent was obtained from all participants. Table 1 presents an overview of the demographic and clinical characteristics of the study sample.

### 2.2. Instruments

Verbal learning and memory was assessed using the German adaptation of the California Verbal Learning Test (CVLT) [30], which was identified and recommended as particularly sensitive to effects of pregnancy [13]. Participants were asked to recall words on a 16-item list (prerecorded and delivered via headphones) after each of the five learning trials. After a distractor list, participants were again asked to recall words from the first list (short-delay recall). Recall was repeated after a 20-min delay (long-delay recall), during which the second test was implemented. According to a psychometric analysis, the number of words recalled in trial 1 primarily relates to short-term memory/attention span; total recall or the progression across trials 1 to 5 is associated with learning efficiency; delayed memory can be assessed by the performance on the short- and long-delay recall trials; and the number of total intrusions displays inaccurate recall [31,32]. Parallel forms were used in test sessions 2 (week 24) to 5 (week 48). The test was implemented with exactly standardized timing, controlled by a computer.

For the assessment of inhibitory functioning, the Mittenecker Pointing Test (MPT) [33] was used, which is unique in its sensitivity also in neurologically intact individuals. Moreover, unlike most other executive tests, such as the WCST, its validity is not affected by repeated application in the same individuals. Rather than providing a diffuse indicator of brain function, the MPT captures specific components of cognitive flexibility [34]. It is a computer-based test requiring participants to press nine unlabeled keys in the most random or chaotic order possible (180 responses in total; for detailed background information on the test and an overview of the validation studies, please see [33,34,35,36]. The context redundancy score (CR), which is based on information theory analysis, specifically captures the capability to inhibit developing routines (the naturally occurring tendency to repeat already selected sequences). CR theoretically ranges from zero (complete absence of any regular pattern) to 1.0 (presence of a fixed, repetitive response pattern, i.e., maximal perseveration). For detailed information on how to compute CR, see Schulter et al. [33].

The Perceived Stress Questionnaire (PSQ; validated German version, 20 items) [37] was used to capture the level of currently experienced everyday-life stress. Scores range from 0 to 60. Sample items are “You feel that too many demands are being made on you”, “You fear you may not manage to attain your goals”, “You feel mentally exhausted”, rated from “almost never” to “most of the time”. Depressive symptoms were assessed by the Center for Epidemiologic Studies’ Depression Scale (CES-D) [38]. It refers to mood and attributions over the past week; scores range from 0 to 33. A rating scale ranging from 0 (never) to 4 (very often) was used to assess mothers’ fatigue.

### 2.3. Statistical Analysis

Data of women with preeclampsia and women with uncomplicated pregnancies obtained at the first measurement time point (16 weeks postpartum) were compared with data of non-pregnant women using one-way analyses of variance. Differences in the progression across trials 1 to 5 of the CVLT were tested with a mixed two-way analysis of variance, with trials as the within-subjects factor and group as the between-subjects factor. Pearson correlations were used to evaluate the relations of experienced stress and depression at the time of testing with learning and memory performance and inhibitory control. Any differences between women with preeclampsia and uncomplicated pregnancies in performance development across the five measurement time points (weeks 16, 24, 32, 40, 48 postpartum) were tested with two-way analyses of variance (within-subjects factor: measurement time point, between-subjects factor: group). Analogous additional comparisons were performed between women with mild vs. severe preeclampsia. The sample size was sufficient to detect medium-sized effects (approx. f = 0.25–0.30) with adequate statistical power (80%) as determined by G*Power.

## 3. Results

No significant differences between groups were observed in any of the cognitive variables, nor in the distress variables. There was a trend toward higher stress levels at the time of testing in new mothers compared to women without a recent pregnancy. Please see Table 2 for a summary of the findings. Additionally performed comparisons between women with mild vs. severe preeclampsia were all non-significant (all *p* > 0.2). Among new mothers, higher levels of experienced everyday-life stress at the time of testing and depression were associated with poorer inhibitory control. Stress levels were not correlated with the learning and memory performance. The correlations are summarized in Table 3.

In the analysis of differences between women with preeclampsia and uncomplicated pregnancies in performance development across the five measurement time points (weeks 16, 24, 32, 40, 48 postpartum), no significant differences emerged (Table 4). Additionally performed comparisons between women with mild vs. severe preeclampsia were all non-significant (all *p* > 0.1).

## 4. Discussion

The findings of the present study do not indicate that the experience of preeclampsia proximately affects verbal learning and memory. In the observation period from 16 to 48 weeks postpartum, women with a history of preeclampsia did not differ from women with uncomplicated pregnancies. Thus, the undisputedly highly stressful experience of a pregnancy complicated by preeclampsia [2,3] per se does not seem to have any impact on mothers’ verbal learning and memory functioning measured from 16 weeks postpartum onwards.

Apart from stress, it is being discussed whether altered vascular functioning might affect brain functions in preeclamptic women [40]. Yet, this seems to be an issue in the rare cases with serious cerebral involvement only [41]. Patients with eclampsia or obvious cerebral symptoms were not included in the present study, but subtle cerebral vascular dysfunction may not be ruled out nevertheless. However, should vascular dysfunction occur in preeclampsia even if a disordered brain function is not apparent 16 weeks postpartum and later, it seemingly does not have a significant impact on cognitive functioning.

The lack of differences in verbal learning and memory performance between mothers with and without history of preeclampsia is in line with the majority of findings in studies that used observation periods in a similar range after gestation and did not report effects of preeclampsia [25,27], with only one exception in a very small sample [26]. The reports of neurocognitive deficits observed later in life [19,20,22] may have suggested effects of preeclampsia. However, considering the lack of rapid-onset impairments, they rather seem to be due to factors acting on a long-term basis, some of which are primarily associated with lifestyle [21].

The finding of unimpaired memory function is somewhat at variance with commonly voiced subjective complaints of cognitive disturbances after childbirth. However, in controlled studies, objectively assessed impairments in new mothers typically fall below subjective memory difficulties, and subjective problems were often not confirmed by objective neuropsychological tests [42]. The crucial difference may be that maximum performance tests are used in laboratory testing, whereas demands in daily life usually do not require women to go to the limits of their capacity. Thus, subjective complaints may not actually be due to declines in cognitive capacity. Instead, the typical performance in the daily lives of new mothers may be affected by factors such as distraction, fatigue, or lack of concentration, which may be attributed to lack of sleep and other circumstances inherent in the lives of new mothers and may to some extent be augmented by the recent experience of a severe pregnancy complication such as preeclampsia.

As opposed to learning and memory, some effects were found for inhibitory functioning, but these were not directly connected with pregnancy complications: higher levels of currently experienced everyday-life stress and depression were associated with poorer inhibitory control. This is in line with previous evidence that high levels of chronic stress are associated with impaired inhibitory functioning, also in the absence of psychopathology [10,11]. The used inhibition measure of the Mittenecker Pointing Test captures the efficiency of a specific inhibitory process, that is, the capability to inhibit developing routines [33,34]. The greater stereotypy in the selection of responses is consistent with the notion that brain functions switch to a mode favoring routine responding during periods of stress [8]. Thus, a minor loss of inhibitory control may occur in women with preeclampsia, if they are experiencing high levels of everyday-life stress. But this is brought about by the general association of stress with a poorer inhibitory functioning, and not by the pregnancy complication as such. Since in the period of observation no differences in current stress levels were found between women with former preeclampsia and women with uncomplicated pregnancies (see Baecke et al. [27] for a similar finding), this association does not automatically put women with a history of preeclampsia at greater risk for inhibition deficits compared to women with uncomplicated pregnancies.

Important, cognitive aftereffects or concomitants of highly stressful events or periods are reversible. For instance, the amelioration of PTSD symptoms over the course of several weeks of therapy goes hand in hand with cognitive and inhibition improvement [43,44]. Thus, stress-related effects should not have a role in the cognitive deficits of women with a history of preeclampsia that are present in later life, many years after gestation [19]—as long as they do not make an impact on unfavorable lifestyles, which often come along with preeclampsia, and are also associated with sustained high stress levels. Stress-related lifestyle factors such as hypertension [45] and poor eating habits [46] may then affect the cognitive functioning of women with former preeclampsia on a long-term basis [21]. It is also important to note that stress and post-traumatic symptoms brought about by the life-threatening condition of preeclampsia alone may be exacerbated in the event of perinatal death.

A limitation of the present study is that women were only tested from 16 weeks onwards, and it was not assessed during pregnancy how threatening the pregnancy complication was actually perceived to be. While it was not feasible in the present study to additionally test similarly well-defined groups of mothers with childbirths complicated or not complicated by preeclampsia some years ago, this is of course desirable in future studies. The sample size, though in the range of, or exceeding, those of related studies in the field, was still at the lower limit. While this may in part be compensated by the particularly well defined exclusion and inclusion criteria and the comprehensive description of the study groups as well as the highly sensitive performance measures, it nevertheless represents a significant limitation. Hence, lack of power should be considered as a potential issue, and the null findings should be treated with caution until replication is available. Related to that, only a minority of women had severe preeclampsia, in which effect sizes and thus statistical power may be greater. Further, future studies may additionally include groups with and without psychiatrically diagnosed post-traumatic symptoms. Another issue of interest in future studies may be to study the moderating effects of other stress-related disorders, such as depression. Depression and anxiety disorders may be particularly important in regard to the long-term development of blood pressure in women with former preeclampsia and, thus, to the unfavorable cognitive outcomes associated with years-long hypertension [47,48]. Similarly, other variables such as body composition, metabolic syndrome, and lifestyle may be important moderators when it comes to the explanation of accelerated cognitive decline later in life [49]. It should also be noted that changes in brain functions during and after pregnancy are not necessarily detrimental to the mother. While there may be some costs, most of these changes represent important adaptations in view of the manifold challenges of motherhood [50]. This may not pertain to additional variations resulting from preeclampsia, though.

## 5. Conclusions

The present study does not suggest a significant rapid-onset cognitive impairment after preeclampsia brought about by its extremely stressful nature or other factors that take effect during gestation. In conjunction with other evidence, it does also not suggest that women with a history of preeclampsia face inevitable cognitive decline in their later life. There is a large time window in which exaggerated cognitive decline, which may develop on a long-term basis through late-diagnosed hypertension and unfavorable lifestyle factors, can be prevented or mitigated. A large proportion of women with former preeclampsia develop hypertension at the age of about 35 to 40 years, which is recognized at only a low rate at this time. When the first diagnosis of hypertension is finally made at the age of about 50 years or later, morphological damage to the vessels, brought about by the years-long pressure stress may already be present [51,52,53]. In addition, radical blood pressure lowering by drugs may produce cerebral malperfusion, which may cause cognitive decline for its part. Taken as a whole, the evidence calls for an increased control of risk factors in the years after preeclampsia, and interventions to improve life style where appropriate [54]. This may also benefit other long-term developments in women with a history of preeclampsia, such as their later cardiovascular health [55].

## Figures and Tables

**Table 1:** Sample characteristics.

	History of Preeclampsia (*n* = 35)	Uncomplicated Pregnancy (*n* = 38)	No Recent Pregnancy (*n* = 40)	Differences between Groups
First measurement time point completed (*n*)	35	38	40	
All 5 measurement time points completed (*n*)	29	35	–	
Preeclampsia: mild, severe	19, 16	–	–	
Age (years)	33.69 ± 4.94 25–42	32.37 ± 4.03 26–44	32.75 ± 5.46 25–44	F_2,110_ = 0.7 *p* = 0.496
**Level of education**				
Less than high school (*n*)	11	7	10	
High school graduate (*n*)	7	9	13	
Some college (*n*)	17	22	17	χ^2^_df=4,n=113_ = 3.4 *p* = 0.492
**Delivery ^5^**				
Gestational age at delivery (days)	253 ± 21 197–287	278 ± 10 254–291		F_1,71_ = 41.5 *p* < 0.001
Child’s height (cm)	46.9 ± 5.1 31–57	51.3 ± 1.8 47–56		F_1,71_ = 24.1 *p* < 0.001
Child’s weight (g)	2568 ± 853 800–3940	3405 ± 336 2780–4010		F_1,71_ = 31.3 *p* < 0.001
Spontaneous delivery (*n*)	6	27		
Cesarean section (*n*)	24	6		
Vacuum extraction (*n*)	5	5		χ^2^_df=2,n=73_ = 24.1 *p* < 0.001
**Life situation ^5^**				
Child’s father in joint household (*n*) ^2^	35	36		–
Support by family members (*n*) ^2^	29	25		χ^2^_(df=1,n=73)_ = 2.8 *p* = 0.097
Breastfeeding at 16 w pp (*n*)	21	30		χ^2^_(df=1,n=73)_ = 3.1 *p* = 0.078
Baby cries at night-time (freq/night) ^1^	1.84 ± 0.82 0.2–3.4	1.85 ± 0.85 0–3.8		t_71_ = 0.1 *p* = 0.940
Baby cries without obvious reason (freq/day) ^1^	1.03 ± 0.43 0–2	1.03 ± 0.55 0–3		t_71_ = 0.02 *p* = 0.987
**Blood pressure**				
Systolic blood pressure (mmHG)^1^	112.5 ± 10.9 88.5–141.1	105.6 ± 8.4 90.7–120.9	110.0 ± 11.1 86.5–140.7	F_2,110_ = 4.3 *p* = 0.016
Diastolic blood pressure (mmHG)^1^	73.8 ± 8.8 58.9–98.6	68.4 ± 6.4 56.3–81.0	71.5 ± 9.3 52.8–89.9	F_2,110_ = 3.9 *p* = 0.024
**Life style**				
Tobacco smoking (*n*) ^3,6^	1	3	10	χ^2^_(df=2,n=113)_ = 9.5 *p* = 0.008
Body Mass Index ^1,6^	27.5 ± 6.1 19.6–44.0	24.6 ± 4.6 18.0–37.8	23.2 ± 3.3 16.9–30.9	F_2,110_ = 8.0 *p* = 0.001
Waist circumference (cm) ^1,6^	94.2 ± 14.7 70–124	88.2 ± 10.9 70–110	79.8 ± 10.2 62–103	F_2,110_ = 13.8 *p* < 0.001
Physical activity (hrs/week) ^1,4,6^	13.2 ± 6.8 2–28	9.3 ± 3.8 3–17	12.2 ± 8.8 1–37	F_2,110_ = 3.2 *p* = 0.044

^1^ Averaged across measurement time points. ^2^ Over entire observation period. ^3^ At any of the measurement time points. ^4^ Total physical activity including everyday physical activities, leisure time activities, and sports activities, assessed by the Freiburger Questionnaire on Physical Activity [29]. ^5^ Variables not assessed in the control group without recent pregnancy. ^6^ Only one measurement time point in the control group without recent pregnancy. Scores of quantitative variables are M ± SD, min–max.

**Table 2:** Verbal learning and memory, inhibitory control, and distress experienced 16 weeks postpartum.

	History of Preeclampsia (*n* = 35)	Uncomplicated Pregnancy (*n* = 38)	No Recent Pregnancy (*n* = 40)
**Learning and memory (CVLT)**			
N of words recalled in trial 1 F_2,110_ = 0.9, *p* = 0.418	7.26 ± 1.48	7.21 ± 1.71	7.65 ± 1.61
Learning efficacy, trials 1–5 Group: F_2,110_ = 0.4, *p* = 0.699 Trial ^1^: F_4,107_ = 414.4, *p* < 0.001 Trial x group ^1^: F_8,216_ = 1.6, *p* = 0.126	7.26 ± 1.48 10.77 ± 2.14 12.83 ± 1.93 13.31 ± 1.47 14.11 ± 1.47	7.21 ± 1.71 10.89 ± 1.83 13.24 ± 2.17 13.97 ± 1.90 14.34 ± 1.51	7.65 ± 1.61 11.37 ± 1.98 12.73 ± 1.77 13.62 ± 1.85 13.93 ± 1.83
Total delayed recall (short + long delay) F_2,110_ = 0.2, *p* = 0.839	26.5 ± 4.06	27.03 ± 4.23	26.5 ± 4.58
Inaccurate recall (n of intrusions) F_2,110_ = 1.1, *p* = 0.342	1.57 ± 2.49	1.08 ± 2.41	0.88 ± 1.16
**Inhibitory control (MPT)**			
Inhibition of developing routines (CR) ^2^ F_2,110_ = 0.04, *p* = 0.964	0.206 ± 0.071	0.205 ± 0.067	0.201 ± 0.078
**Current distress**			
Currently experienced everyday-life stress F_2,110_ = 2.9, *p* = 0.058	16.37 ± 7.29	16.32 ± 7.83	12.85 ± 6.93
Depressive symptoms F_2,110_ = 1.7, *p* = 0.181	9.26 ± 6.95	8.68 ± 6.02	6.80 ± 5.13
Fatigue F_1,71_ = 1.7, *p* = 0.193	1.83 ± 1.2	1.47 ± 1.11	

^1^ Because of the violation of the sphericity assumption, the multivariate approach to repeated measures analyses was used for these effects [39]. ^2^ In the MPT, higher scores denote poorer performance. Scores are M ± SD. We used an alpha level of 0.05 for all statistical tests. Results with *p*-values < 0.05 are considered statistically significant.

**Table 3:** Correlations between currently experienced distress and performance, pregnancy groups, 16 weeks postpartum (*n* = 73).

	Currently Experienced Everyday-Life Stress	Depressive Symptoms	Fatigue
**Learning and Memory (CVLT)**			
N of words recalled in trial 1	r = −0.007 *p* = 0.955	r = 0.027 *p* = 0.823	r = 0.098 *p* = 0.409
Total recalled words, trials 1–5	r = −0.035 *p* = 0.768	r = 0.018 *p* = 0.878	r = −0.014 *p* = 0.908
Total delayed recall	r = −0.012 *p* = 0.923	r = 0.103 *p* = 0.385	r = −0.193 *p* = 0.102
Inaccurate recall	r = −0.149 *p* = 0.207	r = −0.082 *p* = 0.490	r = 0.070 *p* = 0.558
**Inhibitory control (MPT)**			
Inhibition of developing routines (CR) ^1,2^	**r = 0.351** *p* = 0.002	**r = 0.320** *p* = 0.006	r = 0.131 *p* = 0.268

^1^ In the MPT, higher scores denote poorer performance. ^2^ Significant correlations are highlighted in bold font.

**Table 4:** Verbal learning and memory and inhibitory control, weeks 16, 24, 32, 40, and 48 postpartum.

	Week	History of Preeclampsia (*n* = 29)	Uncomplicated Pregnancy (*n* = 35)
**Learning and memory (CVLT)**			
N of words recalled in trial 1	16	7.38 ± 1.45	7.31 ± 1.68
	24	6.81 ± 1.48	6.94 ±1.64
Group: F_1,62_ = 2.2, *p* = 0.143	32	6.41 ± 1.62	7.40 ±1.68
Week: F_4,248_ = 7.3, *p* = 0.005	40	7.38 ± 1.84	7.54 ± 1.70
Week × group: F_4,248_ = 3.2, *p* = 0.163	48	7.28 ± 1.65	8.00 ± 1.37
Total recall, trials 1–5	16	58.59 ± 5.70	59.89 ± 7.36
	24	58.54 ± 6.82	59.11 ± 6.66
Group: F_1,62_ = 1.0, *p* = 0.323	32	59.14 ± 7.26	62.91 ± 6.29
Week: F_4,248_ = 210.6, *p* < 0.001	40	60.35 ± 7.87	60.35 ± 6.86
Week × group: F_4,248_ = 1.6, *p* = 0.176	48	62.83 ± 7.45	64.00 ± 5.11
Total delayed recall (short + long delay)	16	27.07 ± 3.52	26.91 ± 4.35
	24	27.64 ± 3.32	27.40 ± 3.66
Group: F_1,62_ = 0.2, *p* = 0.694	32	29.10 ± 3.52	29.71 ± 2.91
Week: F_4,248_ = 13.5, *p* < 0.001	40	28.59 ± 3.92	26.91 ± 4.70
Week × group: F_4,248_ = 1.8, *p* = 0.132	48	29.48 ± 3.54	29.51 ± 2.31
Inaccurate recall (n of intrusions)	16	1.35 ± 2.45	1.09 ± 2.48
	24	1.20 ± 1.99	0.80 ± 1.50
Group: F_1,62_ = 1.87, *p* = 0.177	32	1.59 ± 1.84	1.11 ± 1.32
Week: F_4,248_ = 4.3, *p* = 0.182	40	0.90 ± 1.35	0.95 ± 1.64
Week × group: F_4,248_ = 0.9, *p* = 0.854	48	0.97 ± 1.59	0.40 ± 0.88
**Inhibitory control (MPT)**			
Inhibition of developing routines (CR) ^1^	16	0.200 ± 0.065	0.204 ± 0.069
	24	0.188 ± 0.045	0.201 ± 0.048
Group: F_1,62_ = 0.7, *p* = 0.406	32	0.184 ± 0.044	0.197 ± 0.042
Week ^2^: F_4,59_ = 1.7, *p* = 0.166	40	0.182 ± 0.044	0.190 ± 0.039
Week × group ^2^: F_4,59_ = 0.4, *p* = 0.784	48	0.184 ± 0.046	0.188 ± 0.039

^1^ In the MPT, higher scores denote poorer performance. ^2^ Because of the violation of the sphericity assumption, the multivariate approach to repeated measures analyses was used for these effects [39].

## Data Availability

The datasets generated during and/or analyzed during the current study are available from the corresponding author on reasonable request.
